# Detection and Analysis of Drug Misuses. A Study Based on Social Media Messages

**DOI:** 10.3389/fphar.2018.00791

**Published:** 2018-07-26

**Authors:** Elise Bigeard, Natalia Grabar, Frantz Thiessard

**Affiliations:** ^1^CNRS, Univ Lille, UMR 8163 STL-Savoirs Textes Langage, Lille, France; ^2^Univ. Bordeaux, INSERM, Bordeaux Population Health Research Center, Team ERIAS, UMR 1219, Bordeaux, France; ^3^DRUGS-SAFE National Platform of Pharmacoepidemiology, France; ^4^CHU de Bordeaux, Pole de Sante Publique, Service D'information Medicale, Bordeaux, France

**Keywords:** drug misuse, patient safety, social media, natural language processing, France

## Abstract

Drug misuse may happen when patients do not follow the prescriptions and do actions which lead to potentially harmful situations, such as intakes of incorrect dosage (overuse or underuse) or drug use for indications different from those prescribed. Although such situations are dangerous, patients usually do not report the misuse of drugs to their physicians. Hence, other sources of information are necessary for studying these issues. We assume that online health fora can provide such information and propose to exploit them. The general purpose of our work is the automatic detection and classification of drug misuses by analysing user-generated data in French social media. To this end, we propose a multi-step method, the main steps of which are: (1) indexing of messages with extended vocabulary adapted to social media writing; (2) creation of typology of drug misuses; and (3) automatic classification of messages according to whether they contain drug misuses or not. We present the results obtained at different steps and discuss them. The proposed method permit to detect the misuses with up to 0.773 F-measure.

## 1. Introduction

According to the existing studies, between 3% Pouyanne et al. (2000) and 20% Queneau et al. (2007) of emergency admissions are caused by adverse drug reactions (ADRs). Other prominent causes of problems related to drugs are drug-drug interactions (DDIs) and drug non-adherence committed by patients. ADRs and DDIs have been studied by researchers (Bate et al., 1998; Bousquet et al., 2005; Duda et al., 2005; Trifirò et al., 2009; Aagaard et al., 2012; Segura-Bedmar et al., 2013; O'Connor et al., 2014; Ayvaz et al., 2015), but issues related to drug non-adherence have been poorly addressed up to now, especially with computational approaches. Yet, in all these cases, the situation is harmful for the patients, who are then exposed to potential safety risks.

According to the WHO, “Adherence to long-term therapy for chronic illnesses in developed countries averages 50%. In developing countries, the rates are even lower” (WHO, 2003). This means that there is a real public health problem of multi-disciplinary nature that shall be addressed. Thus, it has been noticed that *increasing the effectiveness of adherence interventions may have a far greater impact on the health of the population than any improvement in specific medical treatments* (Haynes et al., 2002). Despite the importance of the task, discovery of non-adherence is difficult because patients do not report them spontaneously to physicians or authorities. Hence, the situation is even worse than with the ADRs reporting, which does not exceed 5% (Moride et al., 1997; Lacoste-Roussillon et al., 2001) across the world.

Few works concentrated on the research questions related to drug non-adherence, and they are mainly addressed with manual methods and analyses. Hence, one work proposes to use screening method to detect patients in a non-adherence situation, while specifying that each non-adherence phenotype requires different diagnostic tools (Marcum et al., 2013). However, a meta-analysis performed later did not find that screening methods provide a sufficient tool for the task (Moon et al., 2017). In another work, the researchers conducted an online survey to find out the proportion of individuals in a low adherence situation. It showed that up to 42% users are in this situation. Another purpose of this work was to correlate the non-adherence with other factors, such as being part of an ethnic minority, using multiple healthcare providers and experiencing barriers to access primary care, and this correlation was also confirmed (Feehan et al., 2017). In relation with chronic disorders, some works addressed the non-adherence among patients with diabetes using self-reporting and clinical data (Natarajan et al., 2013). Finally, to collect more exhaustive information, it has been suggested that social media should be used to study non-adherence (Xie et al., 2017).

But even if the exhaustivity can be reached in this way, there is another difficulty. Indeed, it has been observed that non-adherence situations are varied and there's not one solution to fit all patients and situations (Marcum et al., 2013), making more important to study all the ways that non-adherence may appear (Hugtenburg et al., 2013). Moreover, while it is possible to detect non-adherence with traditional methods like monitoring prescription refills, only the users can tell us why they act as such. Only interviews, surveys and social media mining can provide us with such information. Drug misuse is one of those cases, and we will show in this work that it covers several situations as well and that this issue can be addressed with automatic Natural Language Processing (NLP) methods.

Misuses may happen when patients do not follow the prescriptions and do actions which lead to intakes of incorrect dosage (overuse or underuse), to consume drugs for indications different from those prescribed, etc. In order to study drug misuses, we need to use specific sources of information. As has been suggested in other studies (Hugtenburg et al., 2013; Marcum et al., 2013), we propose to focus on information available in health fora: anonymously and without any particular effort, patients willingly talk there about their disorders, treatments, well-being, and actions (Gauducheau, 2008). In this way, it becomes possible to discover some reliable clues about actions and well-being of patients. This information may be useful for medical doctors who can then consider which prevention or information actions are suitable for a given type of patients or drugs.

Very few works are dedicated to the analysis of social media for the observation of drug misuse. We can mention for instance the study of non-medical use of drugs on Twitter. In one work, the researchers used unsupervised machine learning on tweets containing mentions of one of the studied drugs to detect tweets about non-medical drug use (Kalyanam et al., 2017). They also searched for topics discussed by the users, and found out that polydrug abuse was the most discussed topic. In another work, the researchers created a Semantic Web platform for the study of drug abuse on social media (Cameron et al., 2013). The project provided an automatic extraction tool for entities and relationships, and a dedicated ontology based on triples of entities and relationships.

In what follows, we first introduce the objectives of our work (section 2). We then present the material used (section 3) and the steps of the methods proposed (section 4) to reach the objectives. Section 5 is dedicated to the description and discussion of the results obtained, and section 6 draws the conclusion and proposes some issues for future work.

## 2. Objectives

The global purpose of our work is to analyse drug misuses committed by patients. This kind of information is seldom available since patients do not talk about it with their medical doctors and even less with the health authorities. For these reasons, we propose to focus on information available in social media, which provide anonymity to the users as well as the possibility to spontaneously express their experiences. Hence, the objectives of our work are multifold and rely on the use of NLP methods and resources:

- Index social media messages with medical terminologies, providing specific vocabularies used by non-experts in social media, in order to detect drugs and medical problems the patients talk about;
- Propose a typology of misuses, as described by patients in the social media messages;
- Build a language model for the automatic detection of drug misuses.

These objectives guide the design of the methods we propose to follow. This work is done on health textual data written in French. A specific interest is paid to mood disorder drugs, but the methods are generic, this they can be adapted and extended to other disorders and drugs.

## 3. Materials

We use several types of material: a corpus containing messages from discussion fora (section 3.1), a set of drugs (section 3.2), and of disorders (section 3.3). We build the reference data for the evaluation at different steps of the methods. These reference data are described in the corresponding sections of the methods. All the material, processed and built, is available in French.

### 3.1. Forum corpus

We build the corpus from the French health website Doctissimo, and more specifically from two fora dedicated to general questions on drugs^1^ and pregnancy^2^.

Doctissimo is a well-known health platform to the general french public. It is the first thing that comes to the mind of someone with a health question. As such, the contributors are mostly people with punctual questions who are not very familiar with the different health communities on the Internet. This will be reflected in the sort of drugs and illnesses found in our corpus. People with chronic disorders tend to have their own specialized platforms outside of this particular website. There is also simply more healthy people with punctual questions than people with chronic disorders, which means that even if they are individually more active in online communities, the later represent less volume of messages in our corpus.

We collected posts written between 2010 and 2015. We kept only messages that mention at least one drug, for a total of 119,562 messages (15,699,467 words). In each message, the drugs were identified and the drug classes were defined by the first three characters of the ATC codes (as presented in section 3.2). As expected, some drug classes were very frequent. For instance, up to 60% of messages were concerned with birth control pills, and 15% with antidepressant and anxiolytic drugs, which was due to the health concerns of the population and to the topics discussed in the fora studied.

### 3.2. Drugs names

The set of drug names were provided by several sources: commercial drug names and international non-proprietary names associated with their ATC codes (Skrbo et al., 2004), the CNHIM database Thériaque^3^, the *base publique du médicament*^4^, and the database *Medic'AM* from the French healthcare insurance^5^. Thériaque was especially useful because it included short names of drugs typically used by people, such as *Doliprane* / *Tylenol*.

### 3.3. Disorder names

We exploited a set of 29 disorders, for which antidepressants, anxiolytics and mood disorder drugs are used for. This set was created by a medical expert independently from our work. It included terms such as *dépression* (depression), *anxiété* (anxiety), *nerveux* (nervous), *phobie* (phobia), *panique* (panic), or *angoisse* (distress).

Each disorder was associated with the corresponding ICD-10 identifier OMS (1995), such as *anxiety*/*F41* or *depression*/*F32*. The ICD-10 codes are widely used by medical professionals in clinical and research contexts. However, these codes provide a fine-grained and medically-supported difference between the disorders, which patients are usually not able to differentiate. For instance, in the analyzed forum messages, patients usually did not make the distinction between similar diagnoses, such as *agoraphobia*/*F40.0* and *phobia*/*F40*, or *distress*/*F41.0* and *anxiety*/*F41.9*. Hence, the experts were asked to group together such close terms on the basis of their medical knowledge and of indications given by the Word2Vec (Mikolov et al., 2013a,b) clusters. In this way, the most confusing disorders for patients were grouped under simplified codes, such as: *agoraphobia*/*F40.0* and *phobia*/*F40, distress*/*F41.0, anxiety*/*F41.9*, and *generalized anxiety*/*F41.1*. These modifications were done manually by the experts.

## 4. Methods

The proposed methods are composed of several steps: (1) pre-process the material and perform its basic normalization (section 4.1); (2) index the forum messages. For this step, a medical terminology must be adapted to the social media material (section 4.2); (3) propose a typology of the misuses exploiting the content of social media messages (section 4.3); (4) create language models for the automatic detection of misuses (section 4.4); and (5) perform the evaluation of each methodological step (section 4.5). As indicated, all the data used and created are in French.

**Figure 1:**
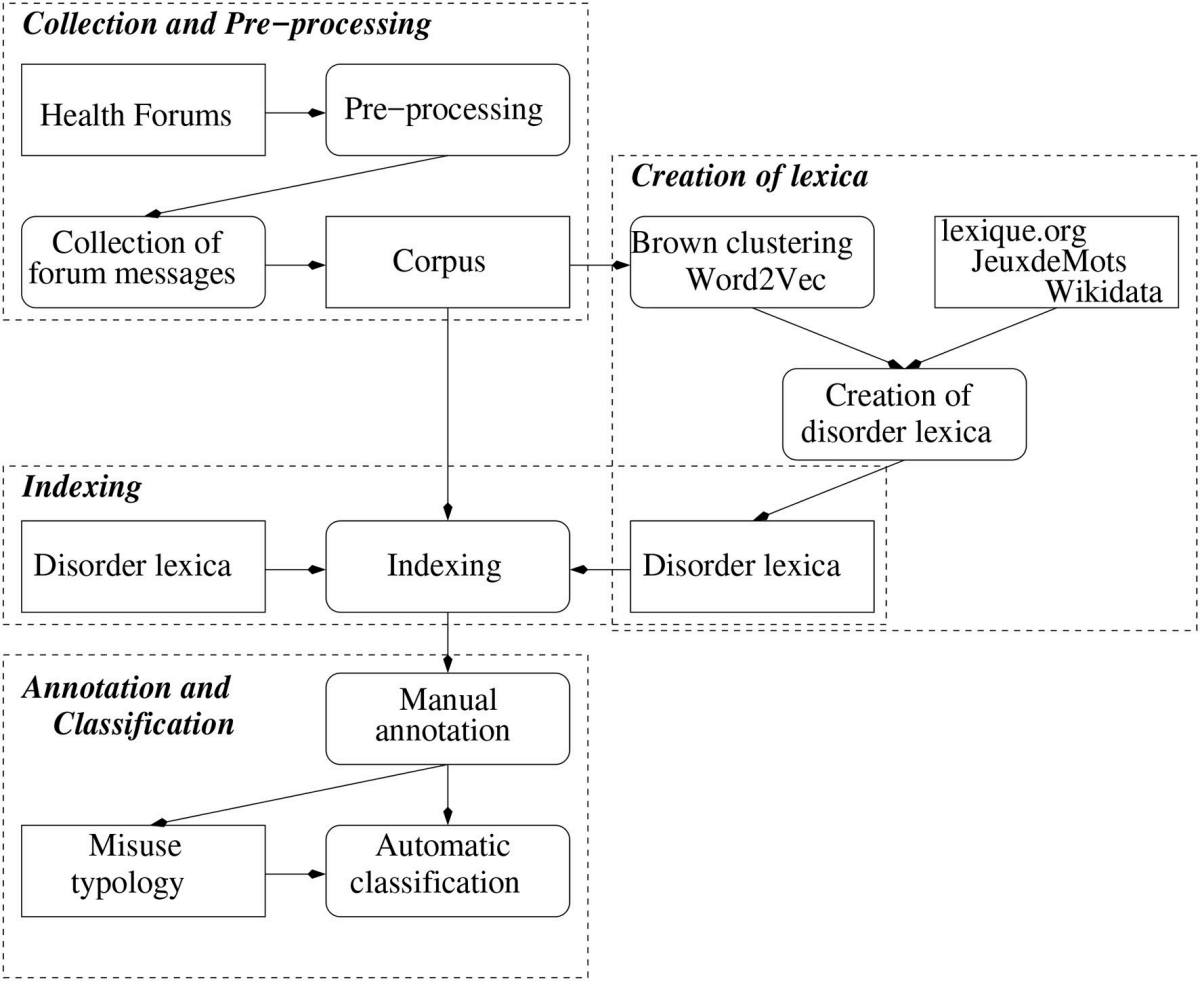
Schema of the steps of the methods.

The way each step of the methods relate to one another is illustrated Figure 1.

### 4.1. Pre-processing of material

The messages were tokenized into sentences and words. The part-of-speech tagging and lemmatization were done by Schmid (1994). This step allowed to assign syntactic information to words (*anxiety/Noun*) and to compute the canonical forms of words {*anxieties, anxiety*}. The numbers were replaced by a placeholder. Diacritics and case were neutralized to lower spelling variations, such as {*Anxiété, anxiete*} (anxiety), in order to allow a further normalization of words. In case of misspellings, the original writing was kept and no spell-checking was performed. As stopwords might be relevant for some steps of the methods, they were not removed from the text.

### 4.2. Indexing of forum messages

The indexing of messages is an important step of the methods, because it permits to mark up drugs and disorders discussed by patients. The main difficulty which we must address during this step is that the terms from medical terminologies, such as ICD-10, are not commonly used by patients. For instance, instead of standard terms like *depression*, patients use equivalent expressions like in this message: *Je ne supporte rien je suis à fleur de peau c'est horrible, je suis hyper nerveuse et obligée de compléter avec une benzo pour me calmer tellement je suisdans un état de nerfs prononcé. (I stand for nothing I am highly strung it's horrible, I am hyper nervous and have to take a benzo as well to calm down I am in a such nervous state*.) This aspect is mainly related to the disorder names, because drug names, being part of medication nomenclature, are submitted to a much lesser variation.

This observation clearly indicates that it is necessary to adapt the used medical terminology and to enrich it with equivalent expressions used by patients in messages posted in social media. Hence, our task consists of building specific lexica related to the disorders we study. We exploited two approaches: (1) the use of existing resources (*Lexique.org, Wikidata* and *JeuxdeMots*), and (2) the use of corpus-driven methods, and more precisely of distributional algorithms (*Brown clustering* and *Word2Vec*). When we used the existing resources, we expected that the needed terms and their synonyms may have been provided by these, although they were created and maintained independently of our work and needs; while the distributional algorithms relied on the content of our corpora and on co-occurrences of words for the generation of clusters with semantically close words, because such words may occur in similar contexts.

The set of the 29 source disorders (described in section 3.3) is referred to as *seeds* in this step of the methods. The existing resources and distributional algorithms were exploited individually or in combination, as presented in sections 4.2.1–4.2.6. The process of automatic indexing is described in section 4.2.7. We also created the reference data (section 4.2.8) for the evaluation of the automatic indexing obtained with these specific resources and algorithms.

#### 4.2.1. Lexique.org

*lexique.org*^6^ is a lexicon built by psycholinguists. It provides links between morphologically related words, such as {*nervous, nervously*}. We used this lexicon to expand our set of seeds through their morphological family: words sharing the same lemma like {*attack, attacks*}, and words sharing their longest morpheme like *nerveux* (nervous) expanded with *nerveusement* (*nervously*) and *nervosité* (*nervousness*).

#### 4.2.2. Wikidata

*Wikidata*^7^ is a semantic knowledge base of general language. It is used to structure the semantic content of Wikipedia and other Wikimedia projects. Several steps were needed before this resource could be exploited for our purpose:

- *Extraction of items that represent disorders*. *Wikidata* contains properties such as *subclass of disease*, but they are not systematically used. Hence, we extracted items associated with the ICD-10 codes corresponding to our seeds;
- *Exploitation of alternative labels*. We also used the property *alternative label* to get various designations for a given item. For instance, the item *agoraphobia* (Q174589) is associated with the French labels *agoraphobe* (*agoraphobic*) and *peur sociale* (*social fear*).

We excluded labels that vary only by the use of diacritics, like {*schizophrénie, schizophrènie, schizophrenie*}, because they were normalized to the same string by our methods in the next steps, but we kept inflectional variants like {*phobie, phobies*} in case of mistakes by the Treetagger POS-tagger and lemmatizer.

#### 4.2.3. JeuxdeMots

*JeuxdeMots* Lafourcade (2007) is a French resource created by crowdsourcing. It contains semantic relations between two words. Each relation is weighted according to how frequently those two words are associated by the annotators. The relationships can be labelled, but most of them are not. We constructed two lexica from *JeuxdeMots*. In one, called *JDM*, we recorded the first 30 related words for each seed, regardless of the relationships. In another, called *JDM morpho*, we kept only words connected to the seeds through morphological relationships.

#### 4.2.4. Brown clustering

*Brown clustering* (Brown et al., 1992; Liang, 2005) is a distributional algorithm designed to create new semantic resources from corpora. On the basis of previous tests, we empirically set the cluster parameter to 500 clusters. In clusters, each word is ordered according to its relevance to its cluster. We first ran the algorithm on the entire corpus, but the resulting clusters appear to be coarse-grained and only few of them contained the seed terms. Hence, we decided to use only a sub-corpus of messages about mood disorders. The new clusters became finer-grained: they contained more relevant terms, and provided more useful content for our purpose.

#### 4.2.5. Word2Vec

*Word2Vec* Mikolov et al. (2013a,b) is another distributional algorithm. It was trained on the sub-corpus with mood disorder messages using the *cbow* algorithm with a 10-word window and bigrams. The evaluation corpus was excluded from the training corpus. This distributional model was exploited for generating clusters for the 29 seeds. For each seed, we kept only the first 30 items. We generated two lexica with this algorithm: *W2V seeds* where the distributional model is queried only with seeds, and *W2V morpho* where the distributional model is queried with seeds and their morphologically related words. At this step, the morphologically related words were exploited in order to decrease the bias of distributional methods to select the words from the same syntactical category as the seed.

#### 4.2.6. Combination

The *combination* of these resources offered two more lexica:

- *Total* contains the merging of all the lexica generated,
- *Vote* contains the seeds, as well as the items provided by at least two distinct resources.

#### 4.2.7. Automatic indexing of messages

Using the various generated lexica, we automatically indexed a given message with a given seed if this seed, or terms associated to it in the lexica, occurred in this message. The same kind of indexing was done at the finer-grained level of sentences.

#### 4.2.8. Creation of reference indexing data

We built the reference data for the evaluation of automatic indexing. This set contains 400 randomly selected messages. These messages were manually annotated by one medical expert. The task consisted of annotating each sentence with one or several disorders from the seeds, if relevant.

### 4.3. Typology of misuses

The purpose of this step is to create a typology of misuses. As has been observed (Hugtenburg et al., 2013; Marcum et al., 2013), patients may commit misuses for different reasons and in different situations. From the clinical point of view, clarification of these reasons and situations allows for more appropriate actions to prevent the misuses. Within the framework of our study, the availability of such a typology is also very useful as it prepares the automatic detection and categorization of misuses. More particularly, it guided the automatic categorization step: first to detect the misuses and, once we collected enough examples, to distinguish the different kinds of misuses.

The creation of the typology of misuses relied on a manual annotation of messages. We first describe the annotation process (section 4.3.1) and then the creation of the reference data (section 4.3.2).

#### 4.3.1. Manual annotation process

The annotator task was to assign each message to one of the following categories:

+ Contains normal drug use, such as in this message: *Mais la question que je pose est 'est ce que c'est normal que le loxapac que je prends met des heures à agir ???* (*Anyway the question I'm asking is whether it is normal that loxapac I'm taking needs hours to do someting???*)
- Does not contain drug use, such as in: *ouf boo, repose toi surtout, il ne t'a pas prescris d'aspegic nourisson??* (*ouch boo, above all take a break, he didn't prescribe aspegic for the baby??*)
! Contains drug misuse. When this category is selected, the annotator is asked to shortly explain what the misuse consists of (overuse, dosage, brutal quitting…). This explanation is done in free text and no previously defined categories are proposed to the annotator. In the following example, the misuse is due to the forgotten intake of medication: *bon moi la miss boulette et la tete en l'air je devais commencer mon “utrogestran 200” a j16 bien sur j'ai oublier! donc je l'ai pris ce soir!!!!* (*well me miss blunder and with the head in the clouds I had to start the “utrogestran 200” at d16 and I forgot of course! well I took it this evening!!!!*)
? Unable to decide.

Three annotators were involved in the annotation: one is a medical expert in pharmacology, the other two are computer scientists familiar with medical texts and annotation tasks. Because this kind of annotation is a complicated task, especially concerning the decision on misuse, all messages annotated as misuse were later reviewed by one of the annotators. Using the short explanation and the content of messages, this annotator verified that the annotation guide was respected.

#### 4.3.2. Creation of reference annotation data

For this step, we exploited the indexing of messages done previously (section 4.3.1) and kept only messages that mentioned at least one drug. Messages with more than 2,500 characters were excluded because they contained heterogeneous information and were difficult to analyse and to annotate. This provided a total of 119,562 messages (15,699,467 words). In each message, the drugs were identified and the drug classes were defined by the 3 first characters of their ATC codes. For instance, G03 covers sexual hormones and N06 antidepressants. As expected, some drug classes are very frequent: up to 60% of posts are concerned with birth control pills, and 15% with antidepressant and anxiolytic drugs.

This set of messages was used to create three corpora that were annotated manually, as explained above:

- *C*1 corpus contained 150 messages selected randomly. Each message of *C*1 was annotated by two annotators independently. This allowed to compute the inter-annotator agreement (Cohen, 1960). In case of disagreement on annotations, the two annotators discussed in order to decide together on consensual annotations;
- *C*2 corpus contained 1,200 messages selected randomly. *C*2 was divided in two halves, each being annotated by one of the annotators;
- *C*3 corpus contained 500 messages. Because some drug classes were more frequent than others, *C*3 was built so that it contained a larger variety of drugs: for each of the 50 most frequent drug classes, we randomly selected 10 posts. We assumed that some misuses could be typical to some drug classes. This motivated the diversification of the analyzed corpus. This corpus was annotated by the pharmacology expert annotator.

All messages annotated as *unable to decide* or as *misuse* were afterwards verified by one of the annotators, and the annotations were modified if necessary.

These three corpora, containing 1,850 manually annotated messages, provided the reference data for our study: on the basis on these annotations we created our typology of misuses, and we also exploited it for the generation of language model for their automatic detection and for the evaluation of the automatic system (section 4.4). This reference dataset provided: 600 messages with no use of drugs, 1,117 messages with normal use, and 133 messages with a drug misuse.

### 4.4. Automatic categorization of misuses

The purpose of this step is to create language models for the automatic detection of misuses in forum messages. We propose to address this problem as a supervised categorization task. We describe here the method designed.

#### 4.4.1. Units processed

Like in earlier steps, the unit processed was the message: it was indexed with disorder and drug names, and it was annotated with drug misuse information from the reference data.

#### 4.4.2. Categories to be found

The objective was to automatically assign the messages into one of the three categories described above (section 4.3.1): + normal use, − no use, ! misuse.

#### 4.4.3. Algorithms

In this work, we used the Weka (Witten and Frank, 2005) implementation of several algorithms for supervised machine learning: NaiveBayes John and Langley (1995), Bayes Multinomial McCallum and Nigam (1998), J48 Quinlan (1993), Random Forest Breiman (2001), and Simple Logistic Landwehr et al. (2005). They were used with their default parameters. The use of these algorithms was combined with the string to wordvector function.

#### 4.4.4. Features

We use several sets of features:

- Lemmatized and vectorized text;
- ATC categories of drugs, identified by the three first characters of their ATC codes;
- ICD-10 codes of disorders identified in the messages.

#### 4.4.5. Experiments

**Figure 2:**
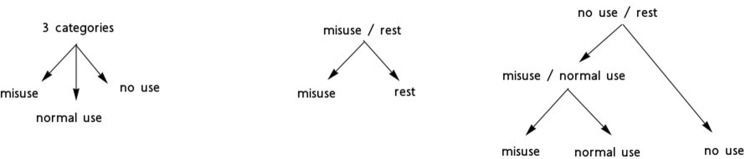
Schema of experiments performed for the detection of messages with drug misuses.

We performed experiments with four sets of language models. The purpose was to detect messages with misuses of medications. Figure 2 proposes the schema of these models and the way these are combined for the detection of the *misuse* messages:

- *Binary categorization misuse-rest*. This model had to contrast the *misuse* category with the two other categories (normal use and no use). The training corpus contained 133 messages from the *misuse* category and 133 messages from the two other categories. This model provided the most straightforward possibility to detect drug misuses in the corpus;
- *Binary categorization no use-rest*. This model had to contrast the *no use* category with the two other categories (normal use and misuse). The training corpus contained 300 messages from the *no use* category and 300 messages from the two other categories. The underlying hypothesis was that the *no use* category may show linguistic specificities compared to the other two categories where the drugs are used, normally or abnormally. This was also a preliminary step toward the following model;
- *Binary categorization normal use-misuse*. This model provided another possibility to isolate the *misuse* messages. It applied to the results obtained with the no use-rest experiment;
- *Tree categories*. The goal was to categorize messages into one of the three categories with the same language model. Since we had 133 messages in the *misuse* category, the two other categories were built so that they contained the same number of messages. This experiment was the most difficult, because the model had to recognize all three categories at the same time.

For each experiment, we used four sets of features:

1. *Text*: lemmatized and vectorized text only;
2. *Drugs*: lemmatized and vectorized text with the ATC codes of drugs added;
3. *Disorders*: lemmatized and vectorized text with the ICD-10 codes of disorders added;
4. *Drugs+Disorders*: lemmatized and vectorized text with the codes from ATC and ICD-10 added.

These sets of features allowed to observe the impact of information on drugs and disorders in comparison with the exploitation of plain text. Besides, in order to better evaluate the impact of the drugs and disorders on the success of the categorization task, we performed two sets of additional experiments, one for drugs and one for disorders, with the following configurations of features:

- *Normal*. The text of messages is used with the original names of drugs and disorders;
- *Code*. The names of the drugs or disorders are replaced by the corresponding codes from ATC or ICD-10;
- *Normal+Code*. The text of messages is used with the original names of drugs and disorders, with the addition of the corresponding codes from ATC or ICD-10;
- *Placeholder*. The names of the drugs or disorders are replaced by a unique placeholder in the text, typically *drug* and *disorder*;
- *Deleted*. The names of the drugs or disorders are deleted from the text.

The reference data used for the automatic categorization of misuses is presented in section 4.3.2. For each experiment, we selected a corpus at random and kept the same corpus for each set of features.

### 4.5. Evaluation

The evaluation of the automatic steps of the methods [indexing of forum messages (section 4.2) and automatic recognition of misuses (section4.4)] was performed with the following measures computed according to the reference data (Sebastiani, 2002):

- True Positives *TP* is the number of correctly classified instances;
- False Negatives *FN* is the number of instances that are not detected by the automatic system although they are expected in the reference data;
- Precision *P* is defined as $\frac{TP}{TP+FP}$ and indicates the percentage of correctly classified instances. When presenting the results, we give the average Precision obtained across all the categories processed in a given experiment;
- Recall *R* is defined as $\frac{TP}{TP+FN}$ and indicates the percentage of correctly detected instances among those expected in the reference data. When presenting the results, we give the average Recall across all the categories processed in a given experiment;
- F-measure *F* is the harmonic mean of Precision and Recall, defined as $2*\frac{Precision*Recall}{Precision+Recall}$.

For the interpretation of the results, the greater the values of Precision, Recall and F-measure, the better the results. The value of True Positives should be as high as possible, while the value of False Negatives should be as low as possible.

The evaluation of the annotation quality, or the inter-annotator agreement, was performed with Cohen's Kappa measure (Cohen, 1960). The measure computes the agreement level between the annotators, given their agreements, disagreements and hypothetical probability of chance agreement. It was suggested that the Kappa results be interpreted as follows: values≤0 as indicating no agreement, 0.010.20 as none to slight, 0.210.40 as fair, 0.410.60 as moderate, 0.610.80 as substantial, and 0.811.00 as almost perfect agreement (Landis and Koch, 1977). Here again, the higher the Kappa value, the better the agreement between the annotators.

## 5. Results and discussion

We present and discuss the results according to the three main steps of the method: indexing of forum messages thanks to specific resources built for social media language (section 5.1); creation of typology of misuses (section 5.2); and automatic recognition and categorization of messages with drug misuses (section 5.3).

### 5.1. Indexing of forum messages

For an efficient indexing of forum messages, the standard terminologies must be adapted to the patient writing such as it occurs in social media. The first step of the methods addresses this point. Table 1 describes the lexica generated from the existing resources and from our corpus. We indicate the size of these lexica, and provide examples of items they offer in addition to the seeds, as well as their translations. The examples are related to the seeds encoded with the ICD-10 code F41: *panique* (*panic*), *anxi* (*anxiety*), and *crise d'angoisse* (*anxiety attack*). The most representative examples for these seeds and for the corresponding lexica were chosen. We can see that additional items are more or less close semantically to the seeds. For instance, *lexique.org*, which contains words from the same morphological family, provides items semantically very close to the seeds, such as *angoissant* (*distressing*) and *angoiss* (*distressed*). *Wikidata* also provides semantically close items, such as *attaque de panique* (*panic attack*). Other resources can provide words that are more distant semantically, such as [*convulsion* (*convulsion*), *crampe* (cramp), *médicament* (*drug*), *alzheimer* (*alzheimer*), *bailler* (*yawn*), *spasmophilie* (*spasmophilia*), *violent* (*violent*), *gros* (*large*), *trembler* (*shiver*), *fesais* (*doeing*)], as well as items that are more relevant for the seeds [*dépersonalization* (*depersonalization*), *hystérie* (*hysteria*), *hystérique* (*hysterical*), *stresser* (*worry*), *suicidaire* (*suicidal*), *cercle vicieux* (*vicious circle*), *devenir fou* (*become crazy*), *spasmo* (*spasmo*), *dangoisse* (*ofanxiety*)].

**Table 1:** Desciption of the generated semantic resources: their size and examples.

**Lexicon**	**Size**	**Exemples**	**Translated examples**
*seeds*	29	*crise d'angoisse*	*anxiety attack*
*Lexique.org*	1,206	*angoissant, angoissé*	*distressing, distressed*
*Wikidata*	83	*attaque de panique*	*panic attack*
*JdM*	20,514	*convulsion, crampe, médicament*	*convulsion, cramp, drug*
*JdM morpho*	69	*angoissant, angoissé*	*distressing distressed*
*Brown*	353	*dépersonalization, hystérie, alzheimer*	*depersonalization, hysteria, alzheimer*
*W2V seeds*	180	*spasmophilie, violent, gros*	*spasmophilia, violent, large*
*W2V morpho*	298	*cercle vicieux, trembler, devenir fou*	*vicious circle, shiver, become crazy*

Table 2 presents the results obtained when the lexica we build are used for the automatic indexing of messages and sentences. For each lexicon and each granularity (sentence or message) we give the following metrics : number of True Positives *TP*, Precision *P*, Recall *R*, and F-measure *F*. This evaluation is done on 400 manually indexed messages. This is our test corpus, since this step of the methods (enriching the lexicon and automatic indexing) is unsupervised.

**Table 2:** Evaluation of indexing on test corpus (400 messages), done at message and sentence levels.

	**Message**				**Sentence**			
*Lexique*	*TP*	*P*	*R*	*F*	*TP*	*P*	*R*	*F*
*Seeds*	297	0.868	0.505	0.639	425	0.779	0.437	0.560
*Lexique.org*	388	**0.881**	0.660	**0.755**	577	**0.801**	**0.594**	**0.682**
*Wikidata*	299	0.869	0.509	0.642	430	0.780	0.442	0.565
*JDM morpho*	339	0.867	0.577	0.693	486	0.778	0.500	0.609
*JDM*	416	0.268	**0.708**	0.389	469	0.113	0.483	0.184
*Brown*	312	0.558	0.531	0.544	436	0.482	0.449	0.465
*W2V seeds*	334	0.536	0.568	0.552	457	0.481	0.470	0.475
*W2V morpho*	338	0.539	0.575	0.557	444	0.450	0.457	0.453
*Vote*	431	0.617	0.734	0.670	618	0.504	0.636	0.563
*Total*	506	0.291	0.862	0.435	696	0.156	0.716	0.256

*The best result for each metric is marked up with a bold font*.

As expected, it is easier to index messages than sentences, because the granularity of information is finer in the second case. At the level of messages, the task is easier because it is possible to find more relevant clues for performing the indexing.

We notice a clear difference in precision values between lexica that usually provide semantically close items (*seeds, lexique.org, Wikidata*, and *JDM morpho*) and lexica that have the tendency to provide words that are more distant semantically (*JDM* and the two distributional methods *Brown* and *W2V*): with the first kind of lexica, precision values are higher. This is not surprising, because morphologically-related words and synonyms are in a better position to preserve the semantics. The best recall is obtained with *JDM*, which is the largest lexicon built. Second best recall is obtained with *lexique.org*, while the third best recall is obtained with *W2V morpho*, built from corpora and morphologically-extended seeds. As for the F-measure values, the best values are obtained with *lexique.org* at the level of message and sentences, due to its excellent precision and one of the highest recall values. If the recall values were to be favored, the combination of lexica *Total* obviously provides the best recall. *Vote* provides high recall as well and the best balance between precision and recall.

The analysis of false negatives indicates that the seed that causes most of them is *dépression* (*depression*), with up to 46% false negatives when using *lexique.org* at the sentence level. We shall notice that this seed was used by the annotator very often, certainly due to its generic meaning. For instance, this term was used to index messages with vague expressions like *un peu moins de joie de vivre qu'avant* (a little bit less of joy to live than before), *baisse de moral* (*decrease of morale*), *je ne me reconnais plus, plus rien de m'intéresse* (*I do not recognize myself, I am interested by nothing*), or *je n'arrive plus à réfléchir ni à imaginer* (*I cannot think or picture things anymore*). This kind of examples clearly indicates that it would be necessary to exploit other methods, for instance supervised learning algorithms, to be able to index and retrieve sentences and messages containing such expressions.

Another interesting observation in relation with the false negatives is related to the fact that the annotator may also have exploited the occurrence of medication names to index the corresponding disorders. Information on intake of medications can indeed be indicative of the fact that someone is suffering from a given disorder. For instance, patients may be looking for advice on drugs without mentioning their disorder, such as in *Qui a eu une amélioration avec cet AD ?* (*Who had an improvement with this AD?*), in which *AD* means *antidepressant*. Typically, this kind of message is not currently indexed for three reasons mainly: (1) it mentions medication instead of disorder, (2) *antidépresseur* (*antidepressant*) is synonym of *dépression* (*depression*) only in two lexica (*JDM* and *W2V morpho*), and (3) the detection of link between the abbreviation *AD* and its expanded form *antidépresseur* (*antidepressant*) may require specific methods.

### 5.2. Typology of misuses

Among the 1,850 annotated messages, 60% (*n* = 1,117) contain normal use, 32% (*n* = 600) contain no use, and 7% (*n* = 133) contain misuse of drugs. The inter-annotator agreement computed on *C*1 is 0.46, which is a moderate agreement. It also indicates that the task on automatic detection and categorization of misuses may be quite difficult. These manually annotated messages are exploited for the development of the typology of misuses.

**Figure 3:**
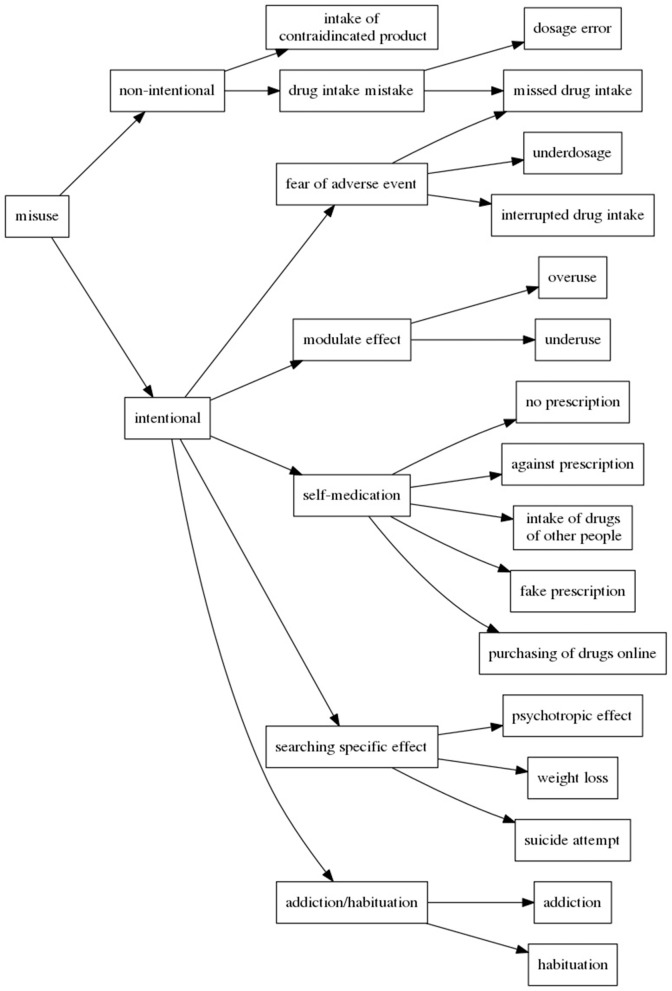
Typology of drug misuses.

In our study of types of misuses, we particularly stress on identifying the reasons leading patients to commit these misuses. Hence, our analysis of the annotated messages indicates that patients can commit misuses of drugs non-intentionally or intentionally (Bigeard et al., 2018). Figure 3 shows the schema of the proposed typology. In case of non-intentional misuse, patients may commit mistakes while taking the drugs, such as intake error, dosage error or being in a contraindication situation. When patients realize their mistake, they usually post a message to ask how to mend the situation. However, when the misuse is intentional, patients do not follow the prescriptions and are aware of it, like in:

- *J'ai arrêté de moi-même (je sais c'est pas bien) (I stopped by myself (I know this is not good))*;
- *j'ai descidé de ne pas en reprendre. (I decided to not take it again.)*;
- *cette fois je rajoute xanax (this time I will add xanax)*.

In other cases of intentional misuses, patients can ignore or overlook the prescription without any particular goal: *ma psy m'a bien dit, pas d'alcool, mais j'en bois quand même en week-end quand y'a des occasions* (*my shrink told me no alcohol, but I drink it anyway during the week-end when I have the opportunity*). Besides, when committing misuses intentionally, patients can: (1) have precise reasons (like the fear of ADRs which leads to underdosage or missed intakes), (2) self-medicate (when they will try to get the prescriptions and drugs they want by any means), or (3) look for particular effects (like psychotropic effect, weigh loss, or even suicide attempt). Yet another misuse situation occurs when patients become addicted to the drugs they take, which mainly happens with anxiolytic drugs. It should also be noticed that sometimes patients may commit misuse intentionally in a moment of temporary distress, which they regret later and worry about the consequences.

From this analysis, we can see that different types of misuses have been detected and that they cover a great variety of situations. We notice that in our reference data, some of misuses occur in very few messages. This indicates that the next step of the Methods, the automatic categorization, will probably illustrate a shortcoming in detecting the less frequent cases.

### 5.3. Automatic categorization of misuses

The results and analysis of the automatic detection of messages with drug misuses are developed through two points: analysis of global results and choice of the best experiments for the automatic detection of misuses (section 5.3.1); and analysis of the role of the drug and disorder names for the automatic detection of misuses (section 5.3.2).

#### 5.3.1. Best experiments for the automatic detection of misuses

**Figure 4:**
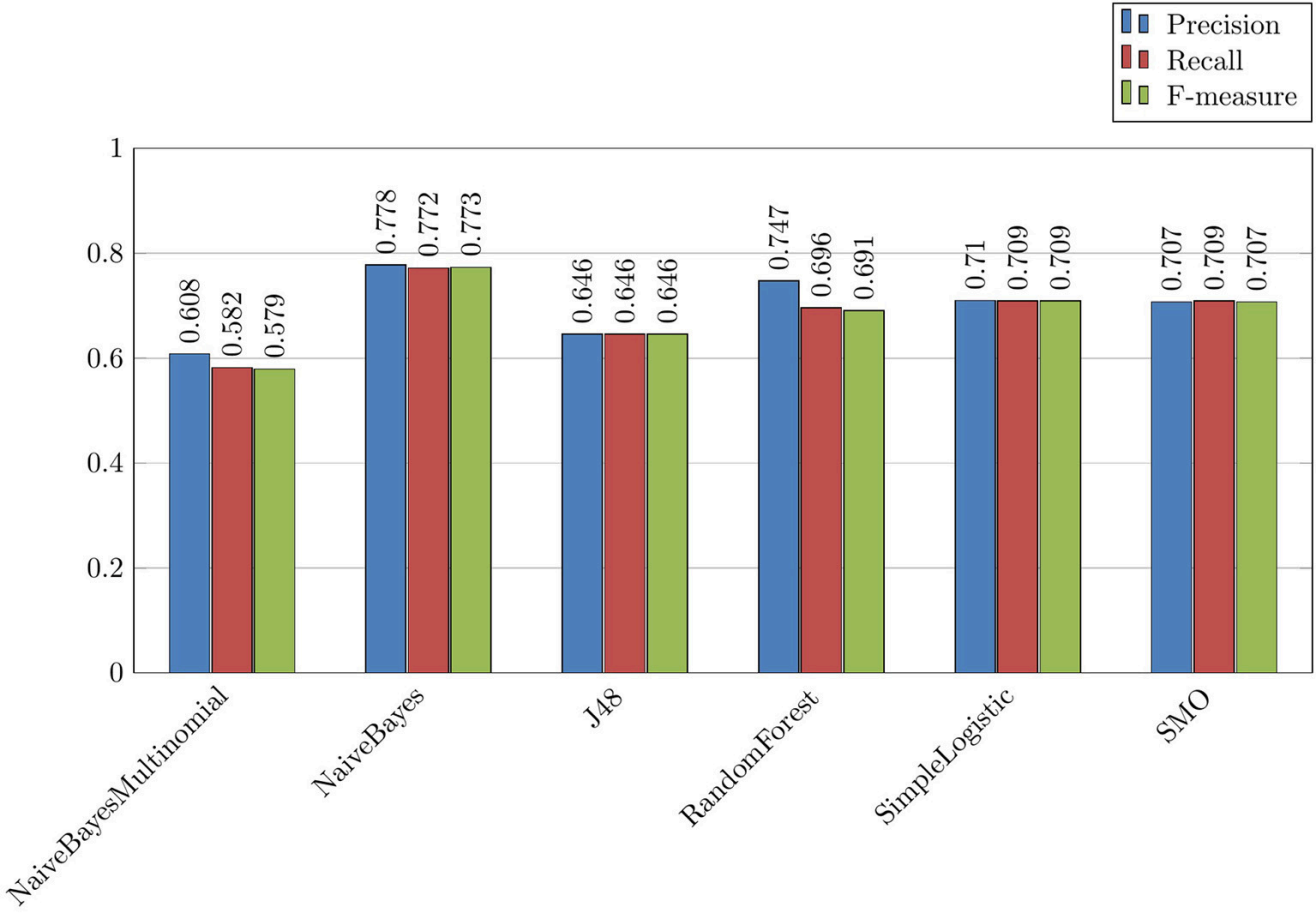
Binary experience *Misuse/Rest* with the *Drugs* set of features and different algorithms.

**Figure 5:**
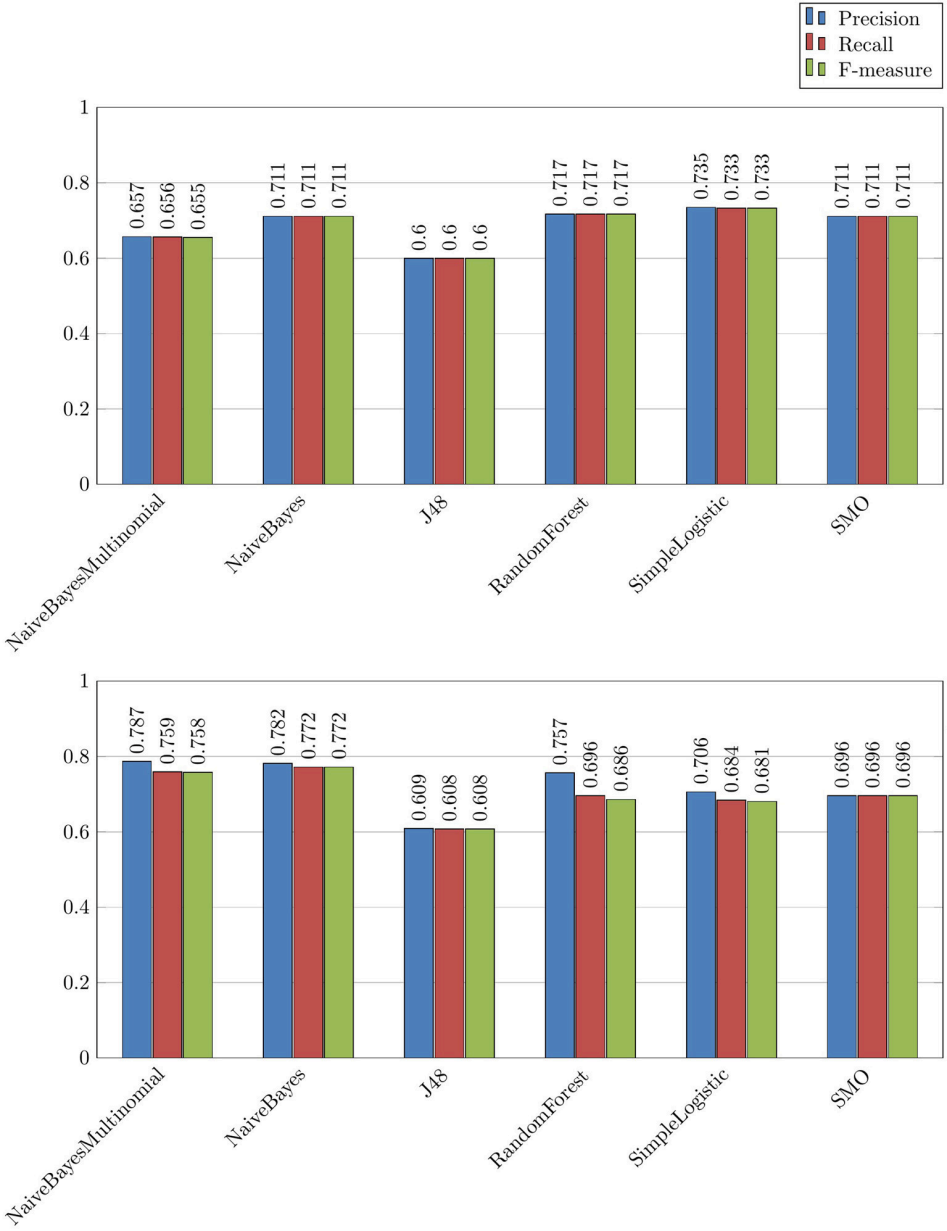
Combined experience *No use/Rest* followed by *Use/Misuse* with the *Drugs* set of features and different algorithms.

We experimented three ways (Figure 2) to detect the messages with drug misuses. For each experiment, we varied the features used (*Text, Drugs, Disorders, and Drugs+Disorders*):

1. *Binary categorization misuse-rest*, which is the most straightforward experiment for the detection of messages with misuses. The results are presented in Figures 4,5. Overall, we can observe that this was the most efficient experiment where we obtained up to 0.773 F-measure with the following parameters: *Drugs* featureset and NaiveBayes algorithm (Figure 4);
2. *Binary categorization no use-rest* followed by *binary categorization use-misuse*, which is a more complicated way to isolate drug misuses because it requires a combination of two experiments. These results are not presented. Here, at the first step we obtained up to 0.733 F-measure, and at the second step we obtained up to 0.772 F-measure. Overall, these results are lower than those obtained with the *binary categorization misuse-rest* model. Several algorithms are competing for the best results. With this configuration, the second step is usually easier to achieve, and the impact of drugs is positive;
3. *Tree categories*, which is an even more complicated way to predict the messages with misuses because this model requires all three categories to be recognized and classified at the same time. As expected, this experiment provides even lower results, with up to 0.613 F-measure.

Overall, we can do several observations on the basis of these results:

1. The *binary categorization misuse-rest* is the most efficient way to recognize the messages with drug misuses.
2. The two most successful algorithms for this task are from the NaiveBayes family (NaiveBayesMultinomial and NaiveBayes). They reach up to 0.773 F-measure (Figure 4). Other algorithms are less successful with the *binary categorization misuse-rest*;
3. Information on *Drugs* (the *Drugs* featureset) has positive effect on the results;
4. The values of precision and recall are usually well balanced in all experiments;
5. Precision values are usually higher than the recall values.

In future experiments, NaiveBayes algorithms should be chosen for the detection of messages with drug misuses.

To understand how the classification algorithms exploit the text of the messages, we perform an analysis of correctly and incorrectly classified messages:

- An analysis of misclassified messages with the *no use/rest* experiment indicates that 27 messages were wrongly classified into the *rest* category and 33 messages were wrongly classified into the *no use* category. Among the incorrectly classified messages, 11 messages do not contain explicit information on the drug intake, such as in this example *elina a quoi pour sa toux ? Ici antibio rebelotte* (*What has elina for her cough? Here antibiotic again*). In 5 other messages, the drugs are not designated by name and are therefore difficult to identify, such as in *j'ai pris mon traitement et les allergies ça va mieux et aussi un spray nasal* (*I took my treatment and allergies are doing better and also a nasal spray*).
- As for the misclassified messages from the *misuse/rest* experiment, we find that 12 messages were wrongly classified into the *misuse* category, and 9 messages were wrongly classified into the *rest* category. Among the 12 messages wrongly classified as misuse, 4 messages contain words that can be associated with excess and harmful effects, such as in *Je n'imaginais pas que c'était si grave* (*I never imagined it was so serious*) or *s'il vous plait ne faites pas n'importe quoi* (*please don't make a mess of things*).
- Finally, the misclassified messages from the *3 categories* classification are distributed as follows: 14 messages were wrongly classified as *no use*, 11 messages were wrongly classified as *normal use*, and 20 messages were wrongly classified as *misuse*. Except the fact that the confusion is higher for the *misuse* category, there is no clear observations on the reasons leading to the wrong classification of these messages.

Overall, this analysis indicates that it is necessary to use larger reference data for improving the quality of the detection of misuses.

#### 5.3.2. Role of drug and disorder names for the automatic detection of misuses

**Figure 6:**
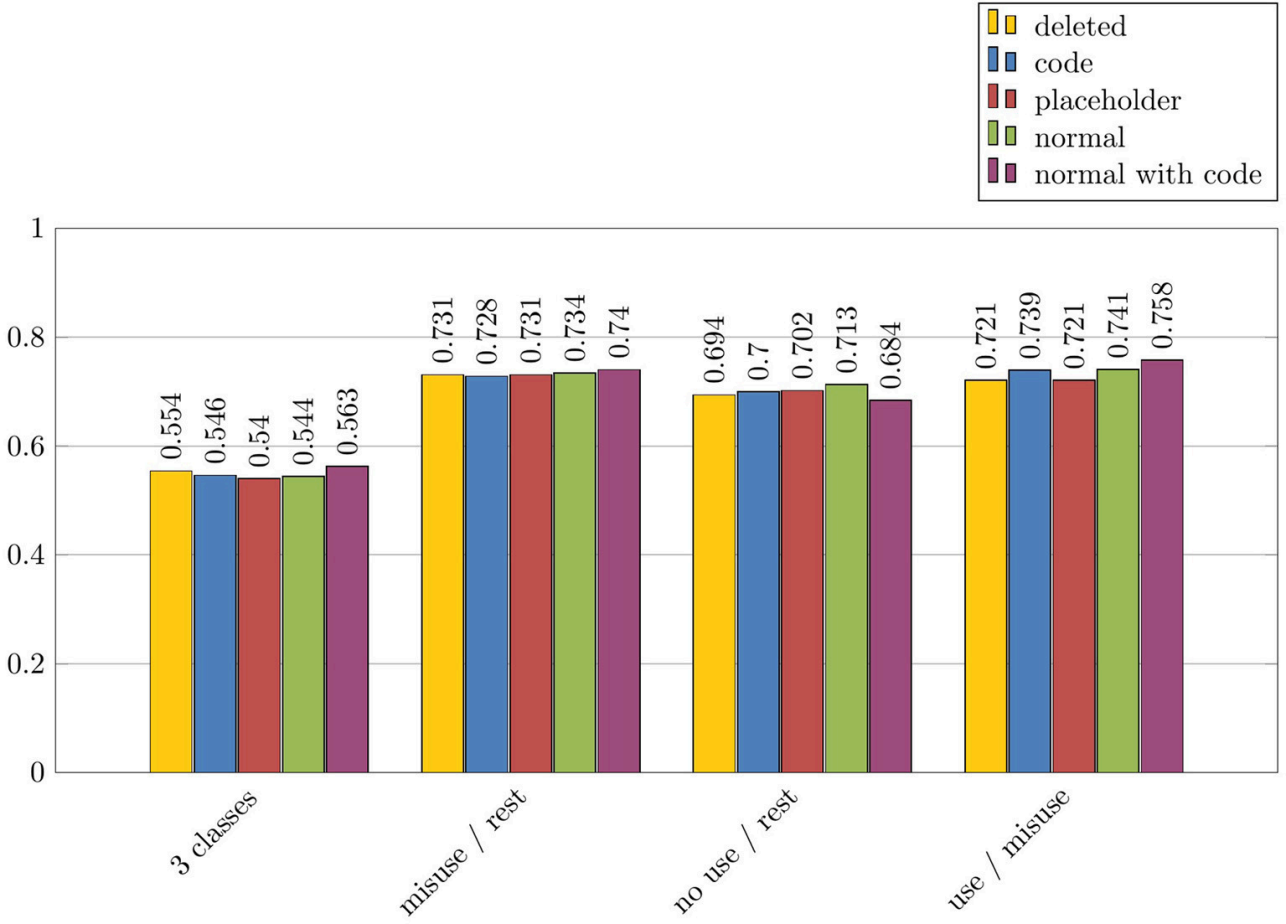
Impact of drug names on automatic categorization results of misuses, in terms of F-measure: *misuse/rest* experiment, *Text* features and NaiveBayes algorithm.

**Figure 7:**
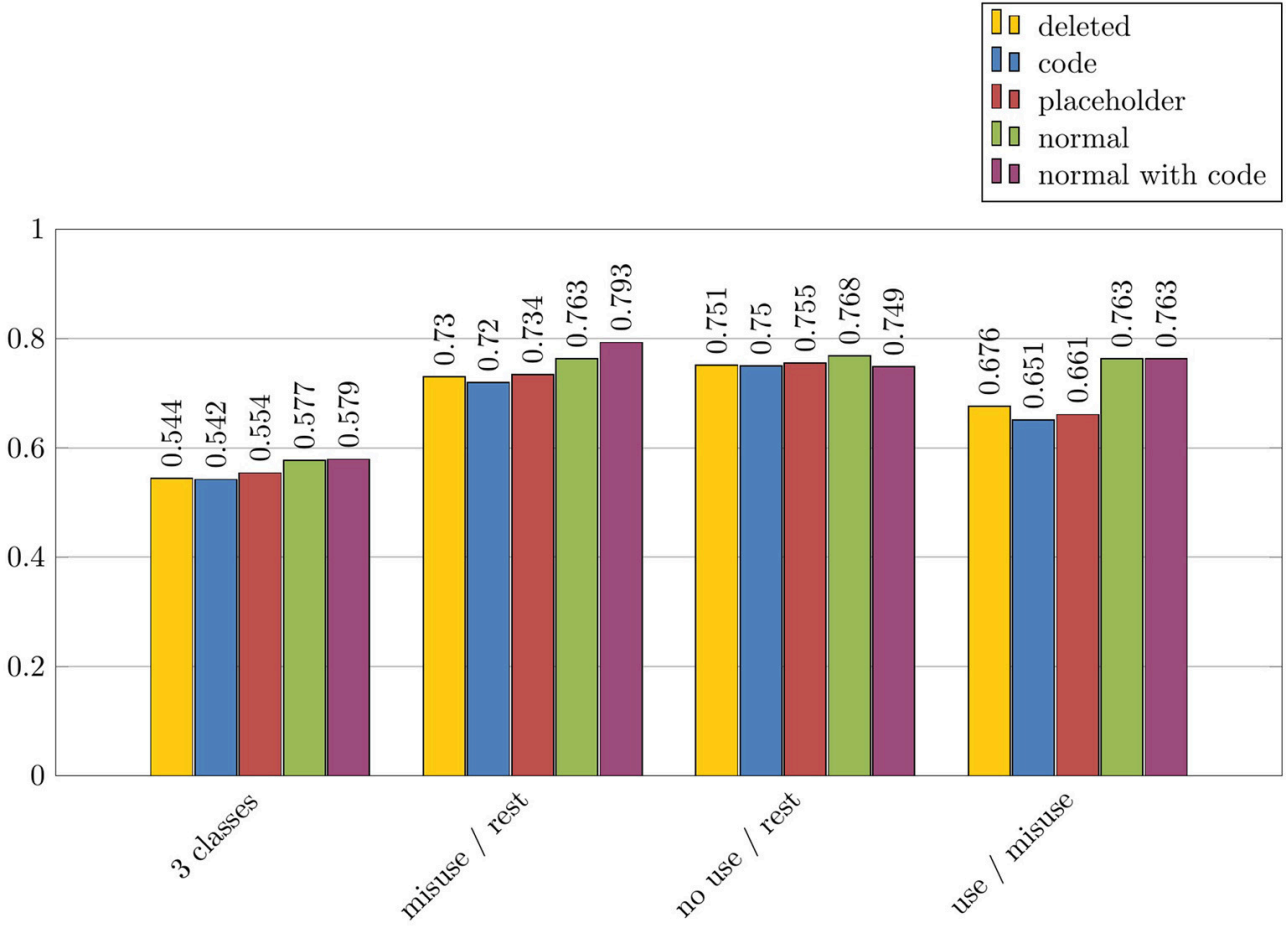
Impact of disorder names on automatic categorization results of misuses, in terms of F-measure: *misuse/rest* experiment, *Text* features and NaiveBayes algorithm.

We performed additional experiments in order to study more precisely the role of the drug and disorder names for the detection of messages with drug misuses. As explained above, five configurations were applied: *Normal* with the original names of drugs and disorders; *Code* where codes from ATC or ICD-10 replaced the names of drugs and disorders; *Normal+Code* with the original names of drugs and disorders, and their codes from ATC or ICD-10; *Placeholder* where the names of drugs or disorders were replaced by the strings *drug* and *disorder*; and *Deleted* where the names of drugs or disorders were deleted from the text. These experiments were all performed with the *misuse/rest* experiment, the *Text* features and the NaiveBayes algorithm. The results for drug names are described in Figure 6, while the results for disorder names are described in Figure 7. These results are indicated with F-measure values.

Overall, we can observe that the results are more impacted by the algorithms and models used, than by the presence of the drug and disorder names. Besides, drug and disorder names show similarly very low impact on the results. For instance, the maximal difference between the results with different configurations is 0.112, and it is lesser than 0.040 for six out of eight experiments. However, we notice small increase of values with the *Normal* and *Normal+Code* configurations, when the names of drugs and disorders remain in the text. Indeed, all experiments show their best results in one of these two configurations, and five out of eight show their two best results in both of these configurations. Furthermore, two experiments with the difference superior to 0.040 between the highest and the lowest results (*misuse/rest* and *normal use/misuse* experiments with the *Disorders* features) are in this position because of a noticeable improvement of results gained with the *Normal* and *Normal+Code* configurations. These observations suggest that the names of drugs and disorders are exploited by the classifiers, even if the difference remains low.

## 6. Conclusion and future work

Our work proposes a set of experiments and analyses that study drug misuses. Though understudied up to now, this is a very important topic as it may provide clues to medical doctors on potential risks related to the prescription of medication. Since the information on drug misuses is difficult to obtain from patients or their relatives, we proposed to exploit discussion fora dedicated to medication and health problems. The work has been done with the French discussion fora from the *Doctissimo* website. This kind of data provides messages naturally produced by the forum users.

To reach the objectives, we proposed to design a method with three main steps: automatic indexing of messages with suitable resources; creation of typology of drug misuses; and automatic detection of messages with drug misuses. We develop the conclusion and future work across these lines.

Patient-authored messages contain specific vocabulary, meaning that we need to use specific resources to be able to automatically index these messages with terms from medical terminology. For the construction of specific resources and enrichment of the terminology, we proposed to use existing resources (*Lexique.org, Wikidata*, and *JeuxdeMots*), and to create new resources using unsupervised corpus-driven methods (distributional algorithms *Brown clustering* and *Word2Vec*). The resources built at this step were used for the automatic indexing of messages and individual sentences. These results were evaluated against the manually created reference data. The results indicate that the resource *Lexique.org* obtained the best F-measure for the indexing of messages, as well as the best precision and recall for the indexing of sentences. The individual resources were then combined. The resource *Vote*, that contained seeds and items proposed by at least two distinct resources, provided one of the best recall without losing a lot in precision. An analysis of content of these resources indicates that the existing resources contain quasi-synonyms and a large number of semantically weakly related words. They may also miss relevant terms. As for the distributional methods, they may collect relevant terms from corpora although often they mix them with more general items, decreasing the precision value. One possibility to improve the results provided by the distributional methods is to exploit bigger corpora for improving the granularity of clusters. Another possibility to improve the quality of resources is to filter the content of clusters, which would allow to select the most specific and relevant items. Rule-based (Grabar and Zweigenbaum, 2005) or probabilistic (Claveau and Kijak, 2014) methods can be exploited for this. We also plan to further improve the exploitation of multi-word queries submitted to the Word2Vec results in order to obtain more specific or more diversified clusters. Finally, this method has been generalized as it has been successfully applied to a larger set of disorders and to other corpora. Currently, two of the resources created in our work (from *Lexique.org* and the resource *Vote*) appear to be the most suitable for the enriching of the medical terminology and for the automatic indexing of forum messages. They can be efficiently exploited for the automatic indexing of messages. Another direction for future work is the maintenance of the resources, which is motivated by two facts: the terminologies used change in time due to the evolution of the domain, and the contents of forum messages change in time due to the evolution of user interests and life. For these two reasons, it is necessary to regularly update these resources for a more efficient indexing and analysis of forum messages.

The purpose of the second step of the methods was to create the typology of drug misuses. We proposed to exploit the content of forum messages dedicated to general questions on medications and pregnancy. For distinguishing the entities of this typology, we relied on the goals of patients when they commit misuses. We identified non-intentional and intentional misuses. On one hand, when the misuse is intentional, patients may want to adjust the effect of a drug by themselves, miss intakes by fear of adverse events, self-medicate, or look for a different effect than the one the drug is prescribed for. In this last case, the motivation can be related to specific psychotropic effects, to weight loss or even to suicide attempts. On the other hand, when the misuse is non-intentional, patients may commit mistakes while taking the drugs (such as intake error, dosage error or contraindication situation). When patients realize their mistake, they post a message to ask how to mend the situation. This typology is built from contents of messages posted on the French health fora dedicated to general questions on medications and to pregnancy, such as available on the *Doctissimo* website. Certainly due to the type of users and questions addressed, some drug classes occur more often than others. For instance, in the forum dedicates to general questions on medication, up to 60% of messages speak about birth control and up to 15% of messages speak about antidepressants and anxiolytics. As for the forum about pregnancy, 44% of messages address only 3 classes of drugs. As explained in the section 4, we tried to overpass this situation and to collect messages that cover a larger set of drug classes. Even if we expect that the drug misuses may show some stable patterns across people and drug classes, there is a potential bias in the data exploited. Because of this bias, currently distinguished types of misuses may be specific to the drugs studied, to the users involved in the fora studied, and to the small amount of the annotated corpus with 133 messages involving misuses. Hence, an additional study is necessary to diversify the corpus with more messages covering a greater variety of drugs and provided by other discussion fora. It is possible that this way, we may discover other kinds of misuses. This is the main direction we plan to take in future work, which we expect will enrich and balance our typology. Yet, it should be noticed that this perspective greatly relies on expertise of the annotators who will be involved in this further work, which is a very heavy task. To help the work of annotators, we can exploit the supervised models for the automatic detection of misuses. Besides, we can also detect and implement specific patterns for the detection of some cases of misuse. For instance, in case of over-uses, we identified recurring linguistic schemes, such as occurrence of quantifiers [*3 boites de xanax* (three boxes of xanax)] or co-occurrence of several medications. As has been observed in previous work, polydrug abuse is indeed highly related to the non-medical use of medications (Kalyanam et al., 2017).

The purpose of the third step of the methods was to design and test automatic algorithms for the detection of messages with misuses. We proposed to exploit supervised classification algorithm for this. Three classes of messages, issued from the manually built annotations and typology, are distinguished (no use, normal use and misuse of drugs), with specific attention paid to the misuse of drugs. This step of the work relies on manual annotation of messages by several annotators, providing the reference data, and on automatic indexing of drugs and disorders, using existing nomenclature and lexica created at previous steps of the Methods. Several experiments are proposed to identify messages with drug misuses. The most efficient experiment distinguishes between two classes: messages with misuses and the rest of messages (no use and normal use). This experiment provides a F-measure up to 0.773. The NaiveBayes family provides the best performing algorithms for this task. Notice that inter-annotator agreement is 0.46, which is a moderate agreement, and indicates that this task is potentially complicated for automatic approaches as well. In addition to the detection of misuses, we proposed to analyse the impact of names of drugs and disorders on the results. Five additional configurations of experiments have been designed: *Normal* with the original names of drugs and disorders; *Code* with codes from ATC or ICD-10 replacing the names of drugs and disorders; *Normal+Code* with the original names of drugs and disorders, and their codes from ATC or ICD-10; *Placeholder* with the strings *drug* and *disorder* instead of real names of drugs or disorders; and *Deleted* with deleted names of drugs or disorders. These additional experiments indicate that the names of drugs and disorders have little effect on the results. Still, when the names of drugs and disorders remain in the text we obtain the best results.

In the general task for the detection of messages with drug misuses, an analysis of misclassified messages points out that the reference data should be enriched to provide a larger variety of messages. As of now, the classification method was only tested on balanced data. It will have to be adapted to the natural distribution of the classes in the corpus. These are the main directions of the future work for improving the quality of automatic detection of messages with drug misuses. We assume that once these limitations are addressed, the proposed methods will the able to be used for the routine detection of messages with misuses.

The proposed supervised models can be used to pre-categorize the messages, for a manual annotation by experts, to enrich the reference data and to make the automatic detection of misuses more efficient. Another direction for the future work may address the automatic distinction of different types of misuses, although this will also require larger volume of reference data. The proposed typology and the detected messages can provide some insights on the reasons that motivate patients to commit misuses of medications. On the basis of these observations and results, specific actions can be taken by medical doctors and pharmacists for the education of patients and for the prevention of drug misuses by them.

## Author contributions

EB performed all the experiments, participated in the annotations, performed the analysis of the data, and participated in the writing of the article. NG participated in the annotations, in the writing of the article, and supervised the work. FT designed the framework of the project, supervised the work, and participated in the writing of the article.

### Conflict of interest statement

The authors declare that the research was conducted in the absence of any commercial or financial relationships that could be construed as a potential conflict of interest.

## Footnotes

^1^http://forum.doctissimo.fr/medicaments/liste_categorie.htm

^2^http://forum.doctissimo.fr/grossesse-bebe/liste_categorie.htm

^3^http://www.theriaque.org

^4^http://base-donnees-publique.medicaments.gouv.fr

^5^https://www.ameli.fr/l-assurance-maladie/statistiques-et-publications/donnees-statistiques/medicament/medic-am/medic-am-mensuel-2017.php

^6^http://lexique.org

^7^http://wikidata.org
